# Editorial: Blood pressure in children and adolescents: Moving forward

**DOI:** 10.3389/fcvm.2023.1142176

**Published:** 2023-01-30

**Authors:** Empar Lurbe, Fernando Fernandez-Aranda, Elke Wühl

**Affiliations:** ^1^CIBER Fisiopatología de la Obesidad y Nutrición (CIBEROBN), Instituto de Salud Carlos III, Madrid, Spain; ^2^Department of Pediatric, Consorcio Hospital General, University of Valencia, Valencia, Spain; ^3^Department of Clinical Sciences, University Hospital of Bellvitge-IDIBELL, University of Barcelona, Barcelona, Spain; ^4^Division of Pediatric Nephrology, Center for Pediatrics and Adolescent Medicine, Heidelberg University Hospital, Heidelberg, Germany

**Keywords:** blood pressure, children, adolescence, hypertension, treatment

Cardiovascular disease to which hypertension (HTN) is a major contributor, has been named as the largest epidemic known to humankind. High blood pressure is a clearly established, modifiable risk factor for early disability and death. Although most of the adverse cardiovascular outcomes occur in adulthood, it has become clear that high blood pressure (BP) is a problem across the course of life that may become evident in early life. Since the 1970s, the prevalence of HTN in children has increased about 4-fold. This data is highly concerning as HTN in childhood is typically associated with intermediate markers of hypertension mediated organ damage (HMOD). As such, further research focusing on early life and assessing the origins of this epidemic is a key issue. The current interest in the field of HTN in youth, could lead to improvements in the quality and efficacy of care provided to patients and lead to secondary prevention of adverse cardiovascular events in later life. There is strong evidence that early identification of high BP and early intervention, can lead to successful management, which has an important impact on long-term cardiovascular health outcomes in adulthood.

The importance of a Research Topic on Blood Pressure in Children and Adolescents can hardly be overemphasized. This Research Topic of Frontiers in Cardiovascular Medicine addresses key issues on the front line of the field of pediatric HTN, highlighting emerging research in the identification, evaluation, and management of high BP in youth, opening the way to make progress on many aspects in the field. Clinical evidence has progressed considerably, and it is now known that HTN in children and adolescents is not at all uncommon and its origin is by no means limited to renal, endocrine, or other diseases but extends to primary HTN which in adolescents and children has a prevalence that makes regular BP measurements mandatory.

The accurate measurement of BP is a prerequisite in children for the reliable diagnosis of HTN and the avoidance of misdiagnosis and over-or under-treatment. In this way Stabouli et al. focused on the named topic as the diagnosis of HTN is critically dependent upon the accuracy of the BP measuring device. The validation criteria for BP measuring devices among consensus documents from different scientific organizations emphasizing on the pediatric population are highlighted and the gaps targeting the needs for validated BP measuring devices are discussed.

The role of primary HTN has gained ground over the last decades, and it is now known that it is the leading cause of childhood HTN, especially in adolescents. The phenotype of primary HTN in childhood and recent findings are discussed in depth by Falkner. For children and adolescents with secondary HTN, the treatment can focus on managing the underlying cause of HTN. Less is known about managing primary HTN in childhood, including diagnosis, evaluation, treatment, and possibilities for prevention.

Evidence has been obtained on the multifold structural and functional abnormalities of the cardiovascular system that can be seen in young hypertensive individuals. Considering its relevance, better knowledge about the natural history of early HMOD is needed. The assessment of HMOD needs to be optimized, looking for early markers in different organ systems. Better knowledge of all of these may contribute to optimize interventions, reducing HMOD and improving long-term prognosis. As summarized by Sinha et al., left ventricular hypertrophy is the main marker of HMOD in children and young people and is evident in at least one-fifth of children and young adults with primary HTN and in nearly a third of those referred to specialty clinics with a predominant eccentric LVH pattern in the latter. Similarly, children and adolescents with primary HTN could be expected to underperform during neuropsychological evaluations when compared with healthy peers. Lucas et al. point-out that evidence relating primary HTN with poor cognitive functioning among youth is usually based on indirect measures of executive functions (e.g., self-reported) rather than objective neuropsychological performance-based tasks. Future prospective studies should consider using common standardized neuropsychological batteries as well as adjust the assessing results for obesity and sleep disorders.

Exercise stress testing as a rather non-invasive procedure to add additional information with regard to cardiovascular risk profile is a relevant tool as expressed by Alvarez et al.. The interpretation of BP values in response to exercise during childhood and adolescence is discussed considering the available reference values and their limitations with regard to device, exercise protocol and normalization. Based on the existing data future studies are needed to extend our current knowledge on possible links between the presence of certain clinical conditions, the detectability of an exaggerated BP response during childhood and adolescence and the risk of developing cardiovascular morbidity and mortality in later life.

A further contribution is devoted to clinical trials with antihypertensive drugs. Redon et al., reinforce that pharmacological treatment in children and adolescents is still limited because there are few randomized clinical trials, hampering appropriate management. Given the increasing prevalence and under treatment of HTN in this age group, innovative solutions including new study designs and optimizing the use of digital health technologies could provide more precise and faster information about the efficacy of each antihypertensive drug class and the potential benefits according to patient characteristics.

The global prevalence of childhood HTN is increasing, yet its investigation has been rather sporadic in Eastern Europe. The calculated prevalence of childhood HTN in Hungary has been assessed by Kovács et al. using data mining methods. Results were comparable to data from other European countries. Higher BMI values were found in hypertensive children as compared to non-hypertensives in all age groups and is associated with early metabolic disturbances.

Furthermore, the relevance of perinatal programming opening up new ways to understand the early-life origins of diseases such as high BP has been covered by Crivelli-Meyer et al.. The authors conducted an inquiry among all employees of public hospitals to assess how many adults remember their own birth weight, an important anamnestic item for cardiovascular and renal disease risk stratification. Waist-to-Height-Ratio is associated with sustained HTN in children and adolescents with high office PB, as presented by Nimkarn et al..

The Research Topic presented here provides an overview of the subject that is difficult to find elsewhere and represents not only up-to-date advice for medical practice but also a source of critical information on issues of great potential interest. We very much hope you enjoy reading this Research Topic.

## Author contributions

EL, EW, and FF-A have all contributed to the design and the writing of this editorial. All authors contributed to the article and approved the submitted version.

